# Supplementary material for: RORα Suppresses Cancer-Associated Inflammation by Repressing Respiratory Complex I-Dependent ROS Generation
Source: Int J Mol Sci. 2021 Oct 1;22(19):10665. doi: 10.3390/ijms221910665 (PMC8509002; doi:10.3390/ijms221910665)
Supplement: Supplementary file 1 [file ijms-22-10665-s001.zip › ijms-1366367-supplementary.pdf]

Supplementary Table S1. Significant differentially expressed genes (from top to bottom)

APOL6	GFPT2	PLCL2	IL6	ADAM8	TINAGL1
AREG	KRT19	TERC	CXCL1	RALB	MOXD1
KCTD2	RGS9	SCD5	MAP3K13	GRAMD3	C12orf28
PDZK1	SAMD9L	TPRG1	FYN	ITGB3	EXOC6B
TTC39B	PLCD4	TMOD2	SRD5A3	CSAD	AKR1C2
ARHGEF6	NCOA	tcag7.1177	PLEKHH2	PHLDB1	PDE4B
STAC	SRPX2	KCTD14	GMFG	TBX15	LYZ
RNASE1	UEVLD	SERINC5	40970	GSN	ANKH
MAN2A2	LHFPL2	RORA	ARNTL	CCNDBP1	ACPL2

Supplementary Table S2. Primers.

Gene	Primer	Sequences (5'-3')
RORα	RTPCR-F	TAACGAATTCGCCACCATGGAGTCAG
	RTPCR-R	CAATGGATCCCCCATCAATTTGCATT
IL-6	RTPCR-F	ACTCACCTCTTCAGAACGAATTG
	RTPCR-R	CCATCTTTGGAAGGTTCAGGTTG
IL-8	RTPCR-F	TTTTGCCAAGGAGTGCTAAAGA
	RTPCR-R	AACCCTCTGCACCCAGTTTTC
IL-24	RTPCR-F	TGTGAAAGACACTATGCAAGCTC
	RTPCR-R	GTGACACGATGAGAACAAAGTTG
IL-32	RTPCR-F	GCTTCCCGAAGGTCCTCTCT
	RTPCR-R	ATAAGCCGCCACTGTCTCCA
Cxcl1	RTPCR-F	GGGGACTTCACGTTCACACTT
	RTPCR-R	GCTCTTCCGCTCCTCTCACA
Cxcl2	RTPCR-F	TCGCACAGCCGCTCGAA
	RTPCR-R	GGGGACTTCACCTTCACACTTTG
Cxcl3	RTPCR-F	TGAATGTAAGGTCCCCCGGA
	RTPCR-R	CACCCTGCAGGAAGTGTCAA
Cxcl5	RTPCR-F	CCACGCAAGGAGTTCATCCC
	RTPCR-R	TTCCTTGTTTCCACCGTCCAA
Cxcl10	RTPCR-F	GTGGCATTCAAGGAGTACCTC
	RTPCR-R	TGATGGCCTTCGATTCTGGATT
NDUFS6	RTPCR-F	TTCGGTTTGTAGGTCGTCAGA
	RTPCR-R	AATGGTGGTGCTGTCTGAACT
NDUFA7	RTPCR-F	GTCCTAGCCACAAGCTCTCC

	RTPCR-R	CACAGGTAAGGCTGGTCCG
NDUFA9	RTPCR-F	TCCTGGGGCGATATGTTGTC
	RTPCR-R	TGGTTTCCCAGTCTCGTCCA
NDUFA11	RTPCR-F	ACAGAGTCACACTCAATCCTCC
	RTPCR-R	AATCCCGTAGTTGTGCGTG
18S rRNA	RTPCR-F	ACCTGGTTGATCCTGCCAGT
	RTPCR-R	CTGACCGGGTTGGTTTTGAT

Supplemental Figure S1

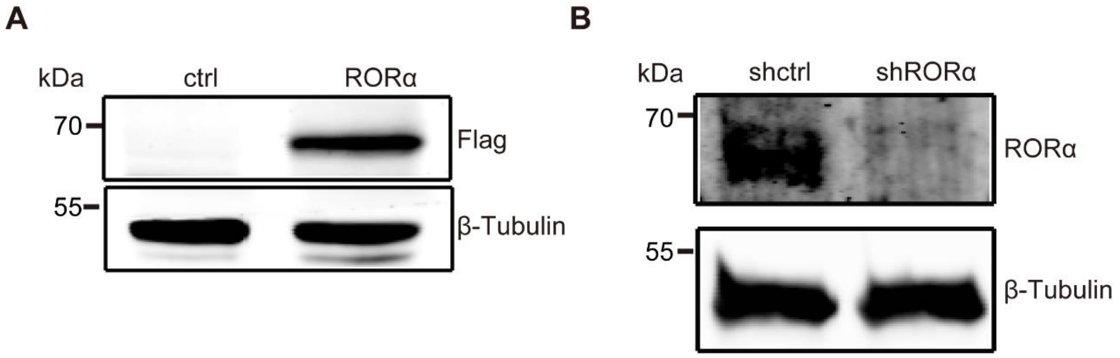

(A) Western blot analysis of flag-tagged RORα protein levels in control and RORα-expressing MDA-MB-231 cells. (B) Western blot analysis of RORα protein levels in control and RORα-silenced MCF-10A cells.

Supplemental Figure S2

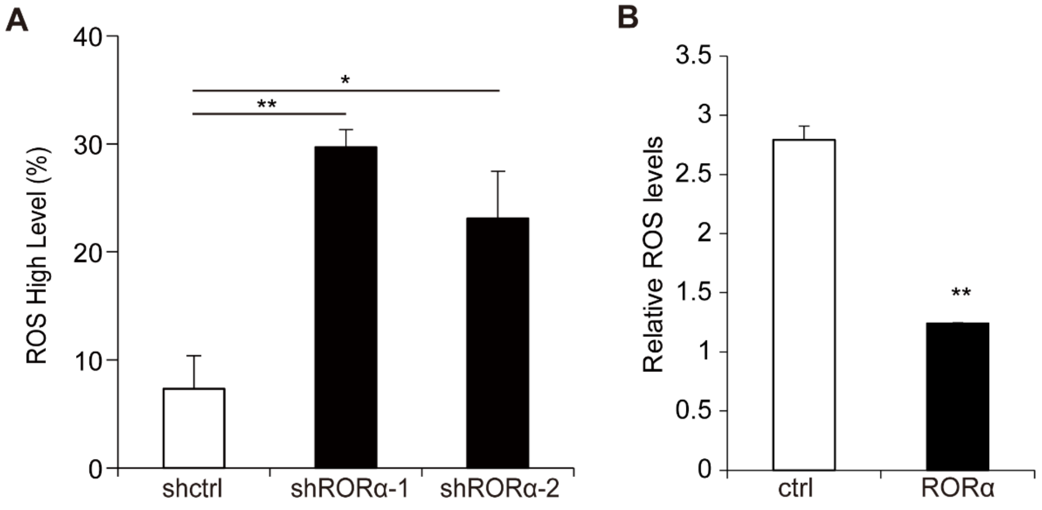

(A) FACS analysis quantified ROS levels in control and RORα-silenced S1 cells; results are presented as mean ± SEM; n=4, ** p<0.01, * p<0.05, one-way ANOVA test.

(B) FACS quantification of ROS levels in control and RORα-expressing MDA-MB-157 cells; results are presented as mean ± SEM; n=4, ** p<0.01, Student's t-tests.

Supplemental Figure S3

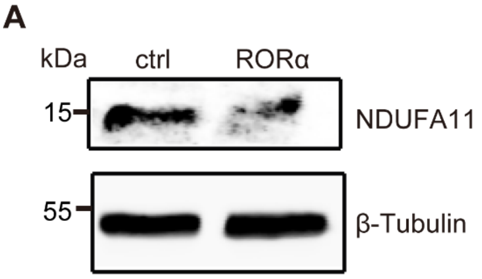

(A) Western blot analysis of NDUFA11 protein levels in control and RORα-expressing MDA-MB-231 cells.

Supplemental Figure S4

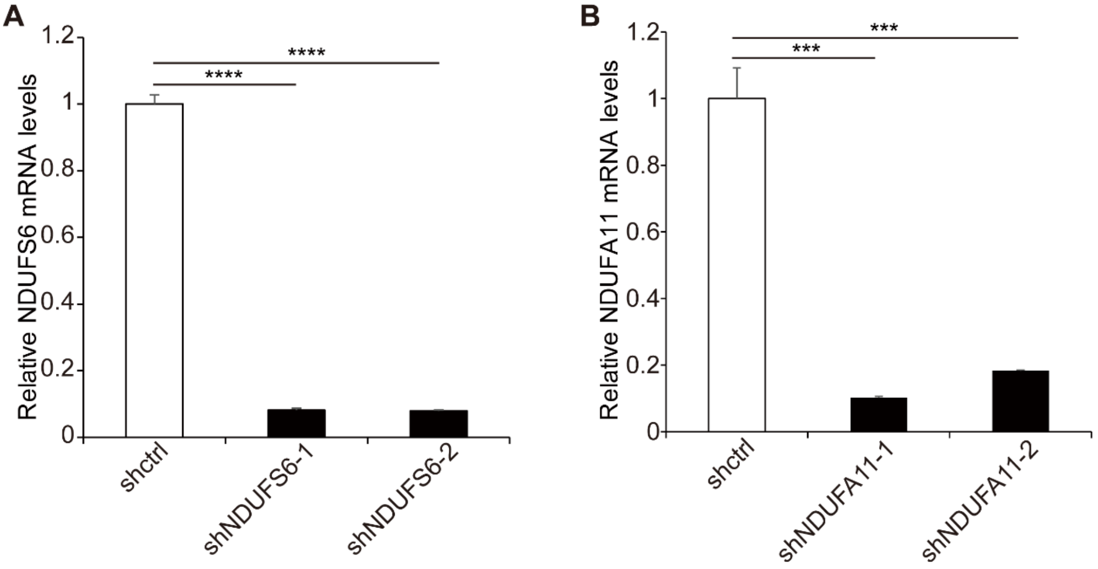

(A) Real-time RT-PCR quantified NDUFS6 mRNA levels in control and NDUFS6-

silenced MDA-MB-231 cells; results are presented as mean ± SEM; n=4, **** $p < 0.0001$, one-way ANOVA test. (B) Real-time RT-PCR quantified NDUFA11 mRNA levels in control and NDUFA11-silenced MDA-MB-231 cells; results are presented as mean ± SEM; n=4, *** $p < 0.001$, one-way ANOVA test.

Supplemental Figure S5

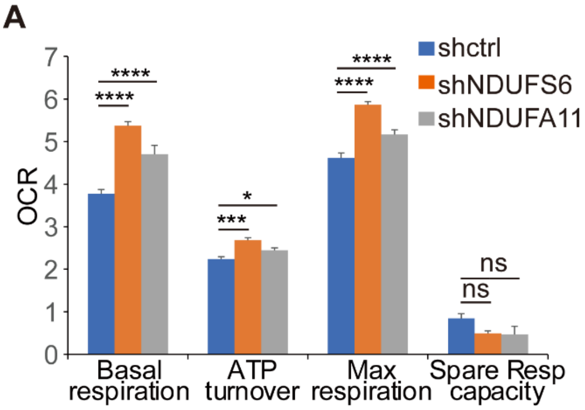

(A) Seahorse analysis quantified OCR in control, NDUFS6-silenced and NDUFA11-silenced MDA-MB-231 cells; results are presented as mean ± SEM; n=12, **** $p<0.0001$, *** $p<0.001$, * $p<0.05$, one-way ANOVA test.

Supplemental Figure S6

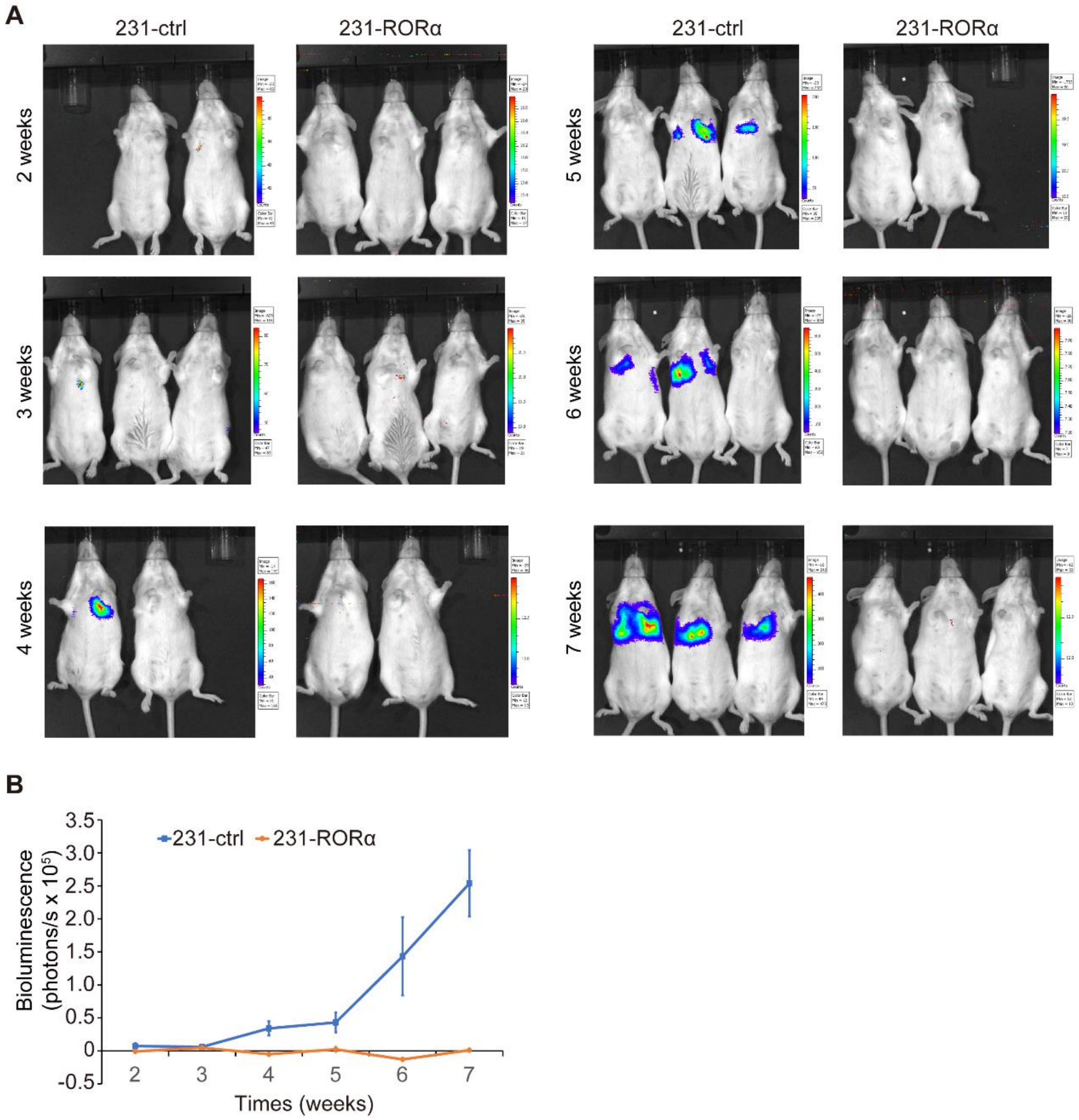

(A and B) IVIS images and quantification data showing lung colonization of control and RORα-expressing MDA-MB-231-luc cells in SCID mice after tail vein injection 2 to 7 weeks; n=6 every group.

Supplemental Figure S7

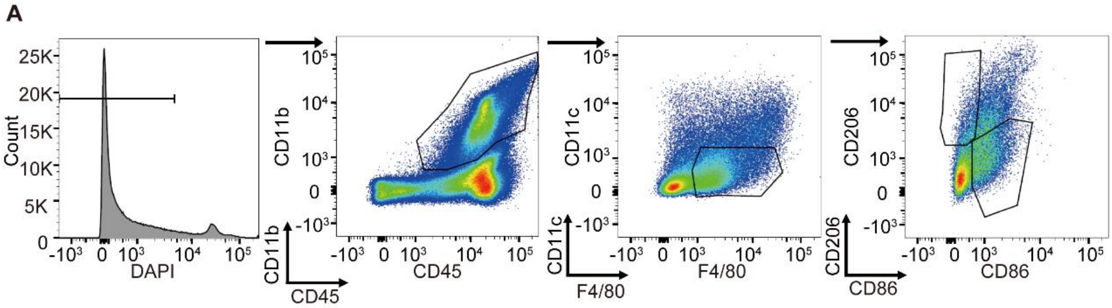

(A) FACS analysis and quantification of macrophages isolated from control or RORα-expressing 4T1 cells injected BALB/c mice. Macrophages were identified as DAPI-, CD45+, CD11b+, CD11c- and F4/80+. Then macrophages were gated for M1 (CD86+CD206-) and M2 (CD86-CD206+).
